# Efficiency of mining rock wastes in the removal of toxic heavy metal ions (Pb²^+^, Cu²^+^, and Cd²^+^) from contaminated water solutions

**DOI:** 10.1038/s41598-026-48461-y

**Published:** 2026-06-04

**Authors:** Aya T. Fathy, Mohamed A. Moneim, Fatma M. Dardir, Abdalla M. El Ayyat, Ezzat A. Ahmed

**Affiliations:** Geology Department, Faculty of science, Assiut University, Assiut, 71516, Egypt

**Keywords:** Phosphatic dolomite (PD), Sodalite (SBPD), Adsorption, Heavy metals, Characterization, Optimum conditions, Chemistry, Environmental sciences

## Abstract

The Abu Tartur Plateau in the Western Desert of Egypt hosts the largest phosphate mining operation in the Middle East. Mining activities in this area generate several million tons annually of overburden waste materials, including carbonate, black shale, siltstone, glauconite, and sandstone. In the present study, phosphatic dolomite (PD) and black shale were collected from these mining wastes. Phosphatic dolomite, along with sodalite-based phosphatic dolomite (SBPD) synthesized from calcined phosphatic dolomite (CPD) and black shale, were evaluated as low-cost adsorbents for the removal of heavy metals (Pb²⁺, Cu²⁺, and Cd²⁺) from synthetic wastewater. Heavy metal contamination, particularly by Cd, Pb, and Cu, represents a major global environmental challenge. Among the available remediation techniques, adsorption is widely considered one of the most effective approaches due to its environmental sustainability, economic feasibility, and operational simplicity. The materials (PD, CPD, and SBPD) were characterized by using X-ray fluorescence (XRF), X-ray diffraction (XRD), Fourier Transform Infrared Spectroscopy (FTIR), Scanning Electron Microscopy (SEM) and BET surface area. Key adsorption parameters—including adsorbent dosage, pH, initial metal concentration, and contact time—were systematically investigated for Pb²⁺, Cu²⁺, and Cd²⁺ removal. The optimal adsorption performance for SBPD was achieved using a dosage of 0.2 g for Pb²⁺ and Cd²⁺, whereas PD showed optimal removal efficiencies at 0.3 g for Pb²⁺ and 0.6 g for Cu²⁺. Both adsorbents exhibited a preferential removal order of Pb²⁺ > Cu²⁺ > Cd²⁺. Kinetic and isotherm models were applied to interpret the adsorption mechanisms. The results of the present study confirm that sodalite based phosphatic dolomite (SBPD) exhibited higher metal removal efficiency than unmodified phosphatic dolomite (PD).

## 1. Introduction

Phosphorus is an essential resource for the production of fertilizers and various phosphorus-based products. As it is neither recyclable nor replaceable, the increasing global demand for phosphorus must be satisfied primarily through the mining, benefaction, and chemical processing of phosphate ores. However, these activities contribute significantly to environmental pollution, as numerous hazardous elements are released into waste streams that may eventually contaminate soil, water, the atmosphere, and food chains^1–3^. Moreover, large-scale phosphate mining operations inevitably generate substantial quantities of waste materials.

Mining waste generally falls into several categories^3^, including:

- Overburden: soil and rock removed to expose the ore body,
- Waste rock: material containing insufficient mineral concentrations for economic recovery,
- Tailing: the residual slurry remaining after ore processing,
- Heap-leach residues: materials left after heap-leaching operations.

**Fig. 1:**
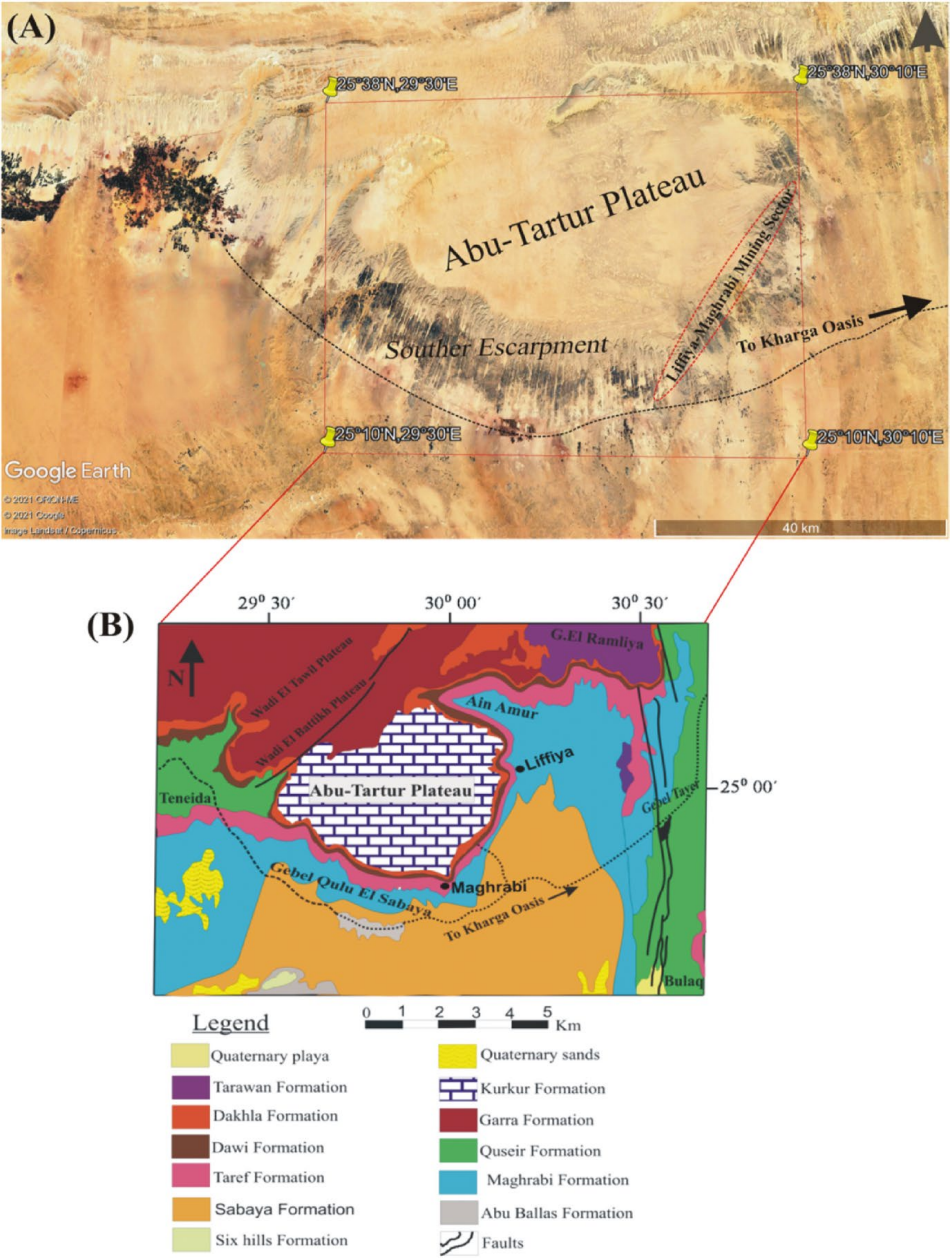
Landsat image showing the location of Abu-Tartur plateau (**A**), with detailed geological map (**B**) from^18^.

In Egypt, phosphate rock represents a major mineral resource, with the country ranking eighth globally in phosphate production in 2020^5^. One of the most significant phosphate mining areas is the Abu Tartur Plateau in the Western Desert (Fig. 1A and B), which hosts the largest phosphate deposit in the Middle East^6^ and contains approximately two-thirds of Egypt’s total phosphate reserves^7^. The phosphate ores in this region are sedimentary in origin and are mainly composed of fluorapatite (Ca₅(PO₄)₃F) and francolite (Ca₅(PO₄,CO₃)₃F)^8^. These rocks commonly contain elevated concentrations of heavy metals such as U, Th, Cd, As, Sb, Pb, V, Cr, Zn, Cu, and Ni^9–11^.

According to internal reports from the Misr Phosphate Company, between 2008 and 2021 approximately 30 million tons of mining waste—equivalent to an annual average of 3.3 million tons—were generated as overburden during operations at the Abu Tartur mine. These wastes mainly consist of carbonate, black shale, glauconite, sandstone, and siltstone. Such materials pose significant environmental risks due to their content of toxic elements (e.g., Cd, Hg, Pb, Cr, Ni, U, Th, and ²²⁶Ra), which may accumulate in soils and leach into surrounding water bodies under weathering conditions, thereby threatening ecosystems and human health^12–14^. In particular, Abu Tartur phosphorites and associated wastes often contain pyrite, which can oxidize during weathering to produce sulfuric acid. This process enhances the dissolution of apatite and facilitates the release of toxic metals into the environment^7,15^.

The recommended permissible limits for Cd, Cu, and Pb in irrigation water and agricultural soils, according to WHO/FAO guidelines^16^, as well as in drinking water according to WHO and USEPA standards^17,18^, are summarized in Table (1).

**Table 1:** Cd, Cu and Pb concentration standards of soil and irrigation water used for agriculture as per WHO/FAO^15^ and drinking water as per the WHO^16^ and USEPA^17^.

Heavy metals	WHO/FAO in mg/L^15^		Drinking water in µg/L/	
	Soil	Irrigation water	WHO^16^	USEPA^17^
Cd	2.25 ± 0.19	0.1	3	5
Cu	28.1 ± 1.8	0.2	2000	1360
Pb	11.31 ± 1.45	5	10	15

Consequently, the effective management and valorization of the large volumes of waste generated at phosphate mining sites have become a major environmental priority. Such approaches aim not only to mitigate the ecological impacts of mining activities but also to explore potential beneficial uses of these waste materials.

Recent studies (2021–2025) have investigated several strategies for utilizing rock wastes generated from the Abu Tartur phosphate mine in various environmental applications. These include:

1. Removal of heavy metals and dyes from contaminated water using untreated or chemically modified mining wastes^19–21^.
2. Development of antibacterial and anti-fungal materials derived from calcined carbonate wastes^22,23^.
3. Adsorption of radioactive technetium (^⁹⁹m^Tc) using organo-clays synthesized from clay and black shale wastes^24^.
4. Biodiesel production using sulfonated carbonaceous bentonite derived from black shale waste^25^.

Building upon these previous efforts, the present study focuses on the synthesis of sodalite zeolite from phosphatic dolomite (PD) waste collected from the Abu Tartur mining area. Both the raw phosphatic dolomite and the synthesized sodalite-based phosphatic dolomite (SBPD) were evaluated for their effectiveness in removing Pb²⁺, Cu²⁺, and Cd²⁺ ions from aqueous solutions simulating contaminated water.

## 2. Materials and methods

All chemicals used in this study were of analytical grade and obtained from commerci.al suppliers. Standard stock solutions of Pb²⁺, Cu²⁺, and Cd²⁺ (1000 ppm) were purchased from Merck and used to prepare synthetic contaminated water samples.

Approximately thirty rock samples of phosphatic dolomite and black shale were collected from the Abu Tartur phosphate mining area (Fig. 1). To avoid the heavy and radioactive metals contamination for the waste rock samples (phosphatic dolomite and shale) which used in the study from phosphatic rocks of Abu Tartor, the weathered surfaces of the used samples were removed before milling.

Aluminum foil with a purity of 99–99.9% served as the aluminum source, while sodium hydroxide (NaOH) pellets used in the modification of phosphatic dolomite were also of analytical grade and commercially obtained.

The characterization of phosphatic dolomite (PD) and the synthesized sodalite-based phosphatic dolomite (SBPD) was performed using several analytical techniques:

- X-ray Fluorescence (XRF): Conducted at Assiut Cement Factory (CEMEX) to determine the chemical composition of the raw materials.
- X-ray Diffraction (XRD): Powder samples were analyzed over a 2θ range of 4–60° using a Philips PW-1710 diffractometer with CuKα radiation operating at 40 kV and 30 mA at Assiut University.
- Fourier Transform Infrared Spectroscopy (FTIR): Spectra were recorded within the 400–4000 cm⁻¹ range using the KBr pellet technique on a Nicolet 6700 FTIR spectrometer at the Chemistry Department, Assiut University.
- Scanning Electron Microscopy (SEM): Selected samples were examined using a JEOL JSM-5400LV scanning electron microscope (Tokyo, Japan) at Assiut University to investigate surface morphology.
- The BET N_2_ adsorption - desorption isotherms of nitrogen at 77 K were constructed using NOVA 3000 Multi-Station High Speed Gas Sorption Analyzer (Quanta- Chrome Corporation), Version 6.07 at Chemistry Department.
- *Atomic Absorption Spectroscopy (AAS): *Concentrations of Pb²⁺, Cu²⁺, and Cd²⁺ in solution were determined using a perkin - elmer AAS instrument at the Geology Department, Assiut University.

### 2.1. Synthesis of zeolite (sodalite)

Sodalite zeolite was synthesized from calcined phosphatic dolomite waste, using black shale and aluminum gel as sources of silicon and aluminum, respectively. The raw materials were finely ground and mixed with NaOH pellets in a mass ratio of 1:1.2. The mixture was subsequently fused in a furnace at 600 °C for 1 h, where NaOH acted as an activating agent.

Following fusion, the solid product was reground and mixed with distilled water at a solid-to-liquid ratio of 1:4.9. The suspension was magnetically stirred for approximately 2 h and then allowed to stand overnight. The reagent ratios used in the synthesis were adopted from Ríos et al^26^.

Hydrothermal crystallization was performed in sealed 150 mL PTFE containers placed inside 200 mL stainless steel autoclaves and maintained at 150 °C for 48 h. After completion of the reaction, the vessels were rapidly cooled using cold water. The resulting solid products were filtered and thoroughly washed with distilled water until the pH decreased below 11, followed by drying in an oven at 80 °C overnight. The dried sodalite samples were then weighed and stored in sealed plastic containers for subsequent characterization and adsorption experiments.

## 3. Results and discussion

### 3.1. Characterization

#### 3.1.1. X-ray diffraction (XRD) analysis

**Fig. 2:**
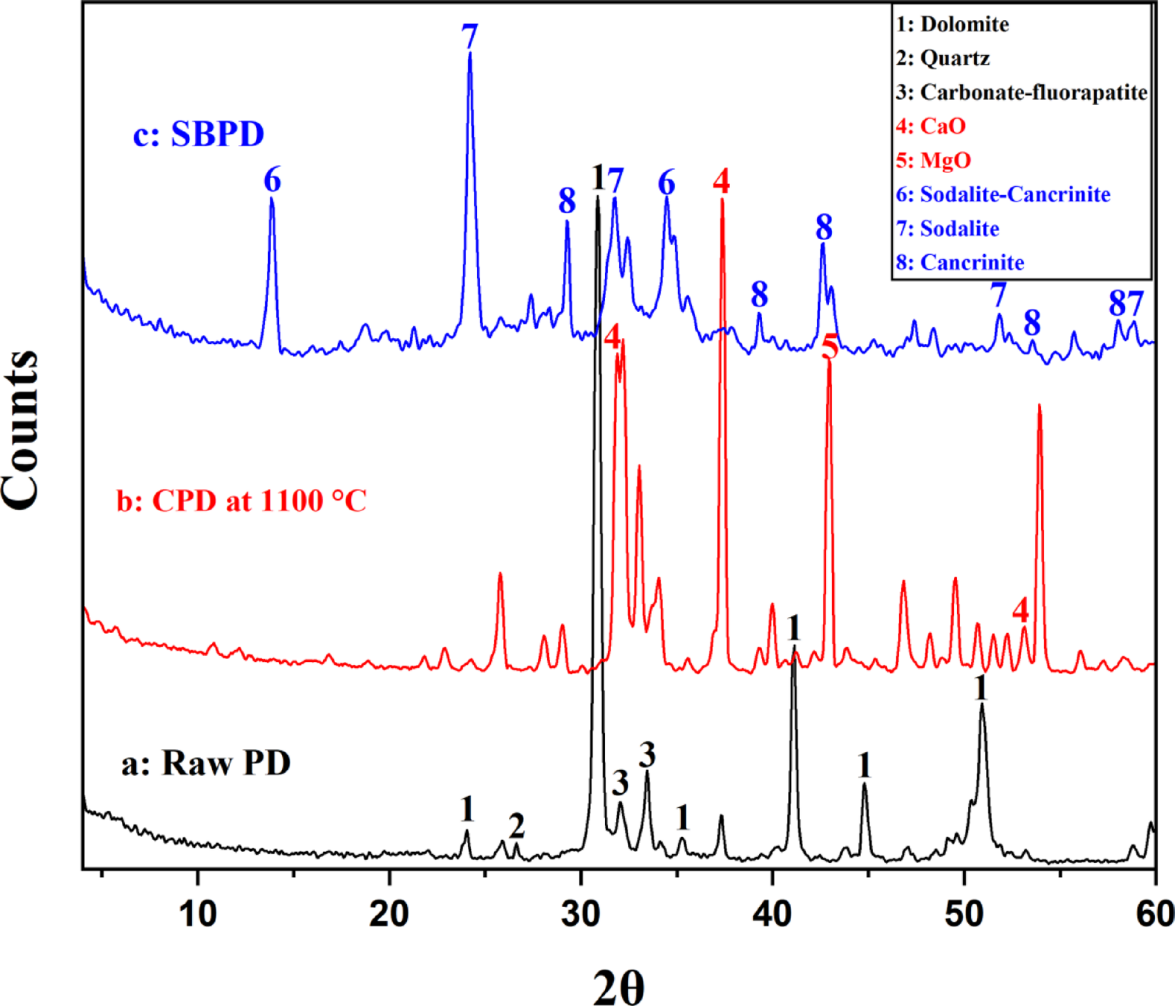
XRD patterns for: (**a**) raw phosphatic dolomite (PD), (**b**) calcined phosphatic dolomite (CPD), and (**c**) synthesized sodalite - based material (SBPD).

The mineralogical composition of phosphatic dolomite (PD) was determined by using X-ray diffraction (XRD) over a 2θ range of 4–60° (Fig. 2a). The XRD pattern of the untreated PD sample displays several distinct diffraction peaks. The most intense reflections correspond to dolomite, CaMg(CO₃)₂, occurring at 24.04°, 30.8°, 35.2°, 41.1°, 44.8°, and 50.9°, which are consistent with the reference pattern of JCPDS No. 96–900−0106. In addition, weak reflections observed at 32° and 33.4° are attributed to carbonate-fluorapatite^27^, while a minor peak at 26.6° indicates the presence of quartz.

Following calcination at 1100 °C, the XRD pattern of calcined phosphatic dolomite (CPD) exhibits the disappearance of carbonate-related peaks, indicating the thermal decomposition of dolomite accompanied by the release of CO₂. The emergence of new diffraction peaks with relatively lower intensity suggests a reduction in crystallite size^28^. Prominent reflections detected at 31.9°, 37.3°, and 53.9° correspond to quicklime (CaO) (JCPDS No. 96–900−6716), while the peak at 42.9° is assigned to periclase (MgO) (JCPDS No. 98-006−0492), as illustrated in Fig. 2b.

For the synthesized sodalite-based phospatic dolomite (SBPD) (Fig. 2c), characteristic diffraction peaks at 24.4°, 31.78°, 52.36°, and 58.84° (2θ) are attributed to sodalite^29,30^. Additional peaks at 13.84° and 34.48° indicate the presence of sodalite–cancrinite phases, whereas reflections at 29.2°, 39.2°, 43°, 53.5°, and 58° confirm the occurrence of cancrinite within the synthesized product^20,31^.

#### 3.1.2. X-ray fluorescence (XRF) analysis

The elemental composition of the phosphatic dolomite (PD) waste rock sample that was determined by X-ray fluorescence (XRF) is presented in Table 2. The rock consists mainly of 35.15% CaO, 10.54% MgO, and 8.97% P₂O₅, along with a loss on ignition (LOI) of 31.33%. The significant P₂O₅ content confirms the phosphatic nature of the dolomitic material. These chemical results are consistent with the mineralogical composition determined by X-ray diffraction (XRD) (Fig. 2a).

**Table 2:** Major oxides analysis of the phosphatic dolomite (PD) sample.

*P*_2_O_5_%	SiO_2_%	Al_2_O_3_%	CaO%	F%	MgO%	SO_3_%	Na_2_O%	K_2_O%	LOI%
8.97	1.52	1.28	35.15	0.91	10.54	4.36	0.27	0.07	31.33

#### 3.1.3. Fourier-transform infrared spectroscopy (FT-IR) analysis

**Fig. 3:**
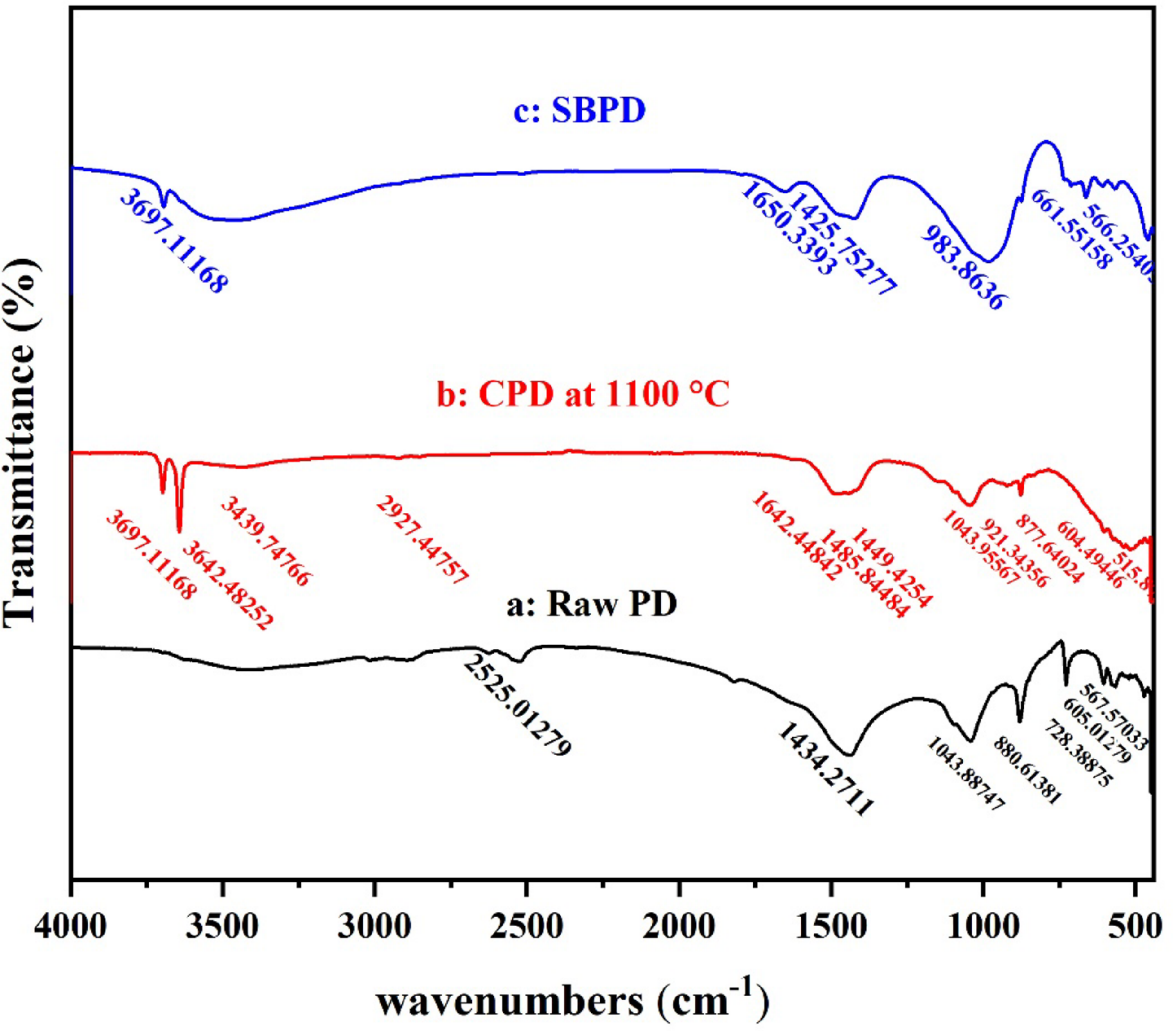
FT-IR spectra of raw PD (**a**), CPD (**b**), and SBPD (**c**).

The FT-IR spectra of phosphatic dolomite (PD) minerals are presented in Fig. 3a. The spectrum shows key vibrational modes of carbonate groups, including the asymmetric stretching vibration at 1434 cm⁻¹, out-of-plane bending at 880 cm⁻¹, and in-plane bending at 728 cm⁻¹, which are characteristic of dolomite^32^. The band at 728 cm⁻¹ corresponds to the bending vibration of CO₃²⁻, while the peak at 1044 cm⁻¹ is attributed to P–O stretching vibrations^33^. Additionally, the band at 880 cm⁻¹ reflects carbonate bending modes. A combination band derived from carbonate vibrations appears at 2525 cm⁻¹, whereas the broad absorption band around 3425 cm⁻¹ is attributed to hydrogen-bonded water molecules^34^.

In the calcined phosphatic dolomite (CPD) spectrum (Fig. 3b), new absorption bands at 3642 cm⁻¹ and 3697 cm⁻¹ appear, corresponding to hydroxyl groups associated with Ca and Mg hydroxides^35^. Bands observed at 1449 cm⁻¹ and 1642 cm⁻¹ are related to symmetric and asymmetric stretching vibrations of O–C–O groups from unidentate carbonate species present on the surface of Ca–Mg oxides. After calcination, broader and stronger bands at 877, 921, and 1043 cm⁻¹ develop, indicating the influence of silicate and phosphate impurities on the dolomite structure^36^. A band at 3439 cm⁻¹ again suggests the presence of hydrogen-bonded water molecules^34^.

For the sodalite based phosphatic dolomite (SBPD) spectrum (Fig. 3c), characteristic zeolite-related bands are observed. The asymmetric and symmetric Al–O stretching vibrations occur at 983 cm⁻¹ and 661 cm⁻¹, respectively^37,38^. Water-related OH stretching and bending vibrations appear at 3697 cm⁻¹ and 1650 cm⁻¹, indicating the presence of sodalite and sodalite–cancrinite phases^39–41^. A strong ν₃ carbonate band at 1425 cm⁻¹^42^ and a PO₄³⁻ bending vibration at 566 cm⁻¹^43^ further confirm the complex composition of SBPD.

#### 3.1.4. Scanning electron microscope (SEM) analysis

**Fig. 4:**
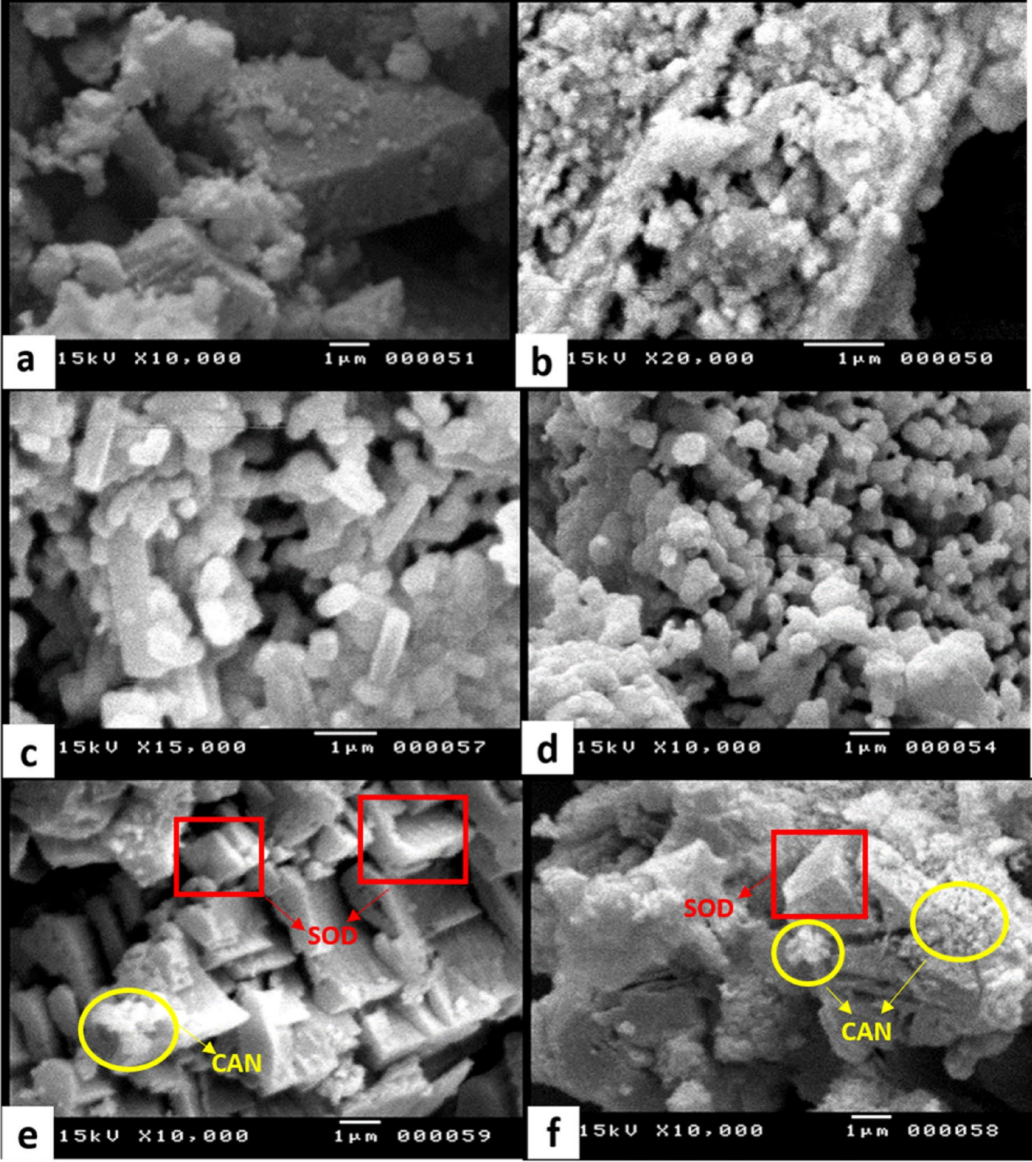
SEM micrographs of PD (**a**, **b**), CPD (**c**, **d**), and SBPD (**e**,** f**), showing changes in morphology and crystal development through calcination and synthesis.

The morphological characteristics of raw PD, CPD, and synthesized zeolite (SBPD) were investigated by using scanning electron microscopy (SEM) (Fig. 4). Figure 4a and b show that PD exhibits a blocky morphology with variable particle sizes and sharp edges. The surface appears rough and uneven, with a dense rhombohedral structure.

After thermal treatment, CPD displays a more fragmented texture. Calcination causes the decomposition of dolomite into fine particles of CaO and MgO, releasing CO₂ and generating surface voids^44^. As a result, the surface becomes more rugged and the particle size decreases. CaO typically forms rounded particles, whereas MgO aggregates appear in cubic or clustered morphologies (Fig. 4c and d). These changes enhance the surface area and porosity.

Following the synthesis of SBPD by incorporating black shale into CPD, sodalite crystals are formed. SEM images (Fig. 4e and f) show well-defined cubic crystals. Spherical cancrinite crystals, known as “lepispheres,” appear as intergrown thin disks developed from wedge-shaped or isometric sodalite crystals. Such phase transformation from sodalite to cancrinite has been previously reported^45,46^.

### 3.2. N_2_ adsorption - desorption isotherms

**Fig. 5:**
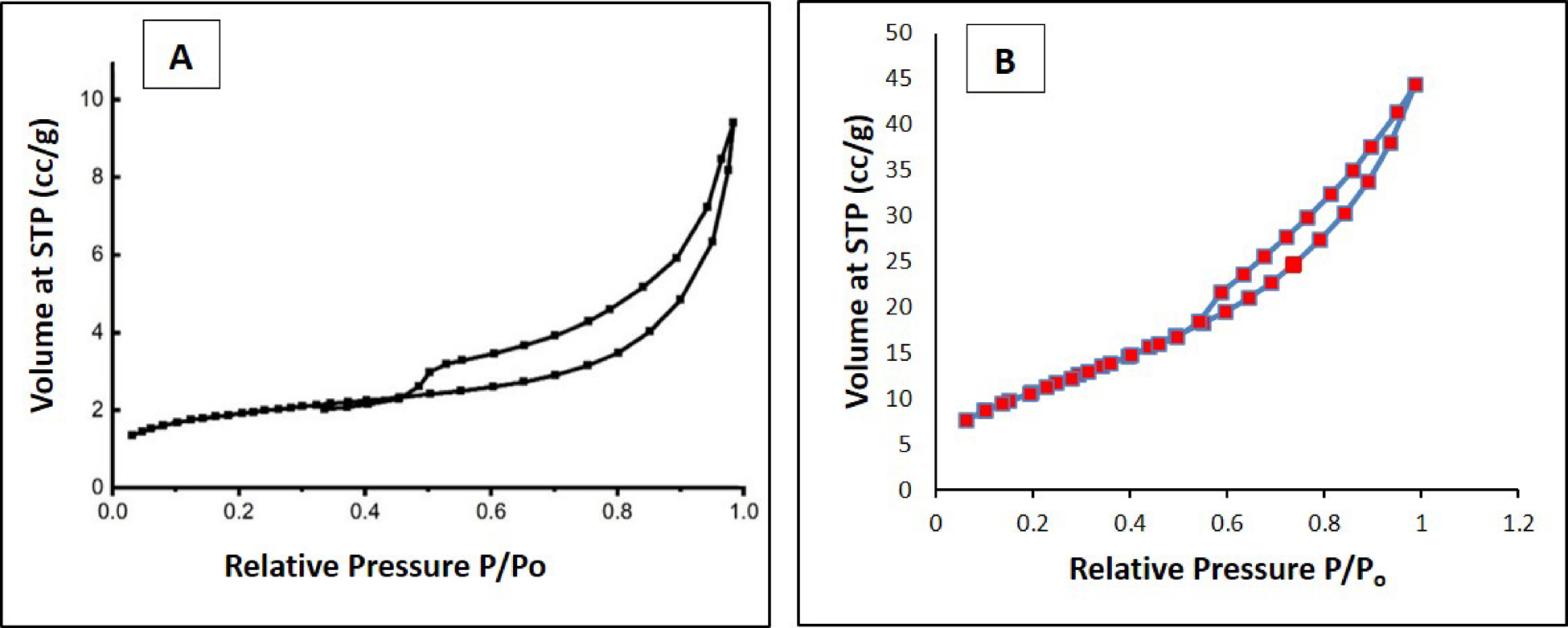
N_2_ isotherms for PD (**A**) and SBPD (**B**).

Surface characteristics of PD and SBPD samples were determined by BET-method using low temperature nitrogen adsorption- desorption isotherms (Fig. 5). Both samples) are exhibit a type IV isotherms and hysteresis loops value 0.5–1.0.5.0 (P/P0) caused by capillary condensation of nitrogen. These are a characteristics of mesoporous materials^47^^48^,, The estimated pore size was 44. 5 Aº for PD and 47.3 Aº for SBPD sample. Surface area as well as pores volume calculated by BET analysis were 6.8 m^2^/g and 0.092 cc/g for PD sample and 40.7 m^2^/g and 0.164 cc/g for SBPD sample.

### 3.3. Adsorption results

**Fig. 6:**
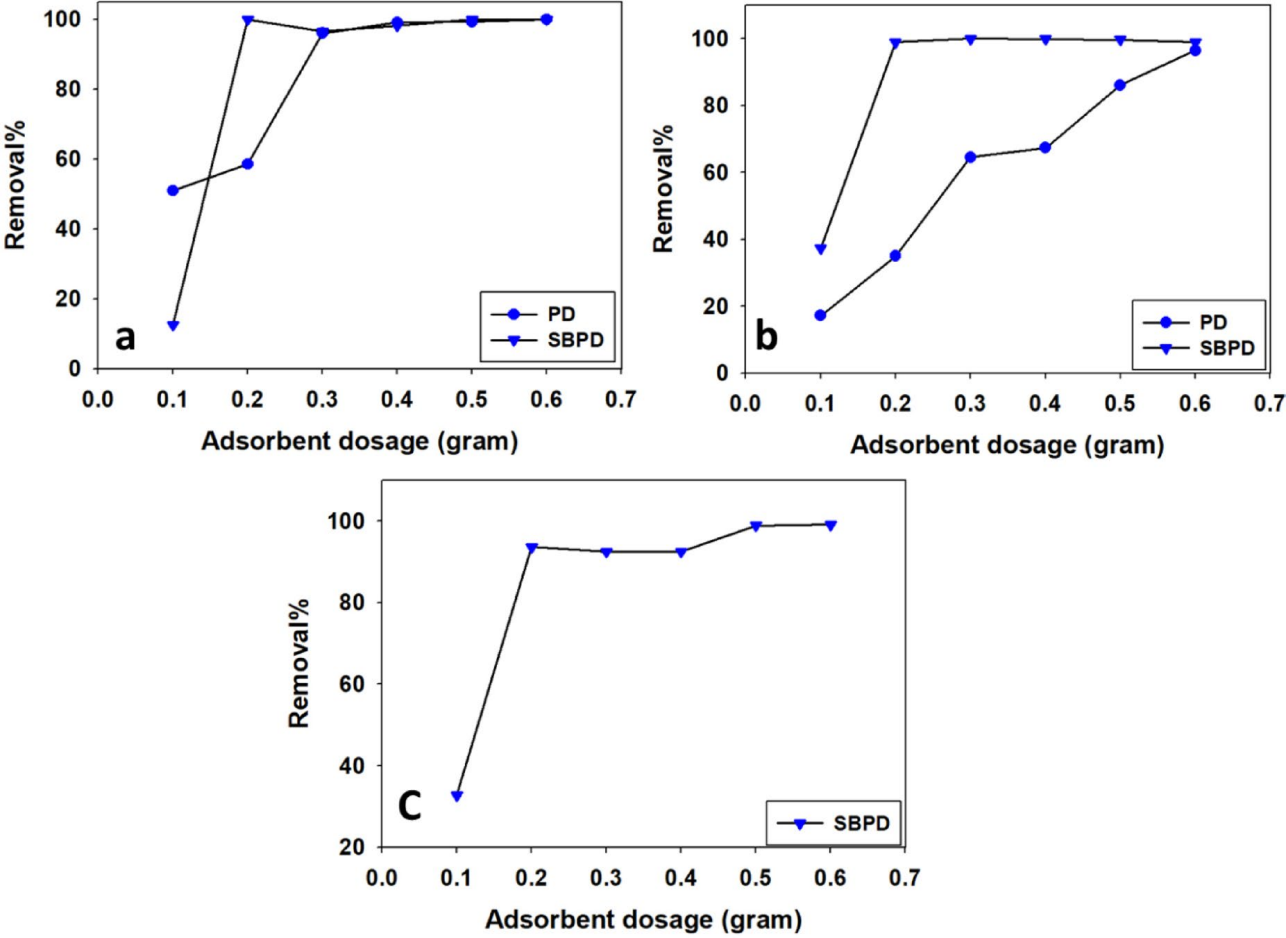
The optimum sorbent dosage for PD and SBPD to removal of Pb^2+^ (**a**), Cu^2+^ (**b**), and Cd^2+^ onto SBPD (**c**).

The adsorption efficiency of phosphatic dolomite (PD) and synthetic sodalite derived from phosphatic dolomite (SBPD) for the removal of heavy metal ions (Pb²⁺, Cu²⁺, and Cd²⁺) from aqueous solutions was evaluated. Key parameters including adsorbent dosage, pH, contact time, and initial metal ion concentration were investigated to determine the optimal conditions for maximum adsorption. The results are illustrated in Figs. 6, 8, 9 and 9 and summarized in Table 3.

**Table 3:** The optimum conditions for the removal of Pb^2+^, Cu^2+^, and Cd^2+^ ions onto phosphatic dolomite and sodalite adsorbents.

Heavy metal	Optimum Conditions	Phosphatic dolomite (PD)	Sodalite (SBPD)
Pb^2+^	Adsorbent dosage (gm)	0.4	0.2
	pH	3	3
	Initial concentration (ppm)	350	1000
	Contact time (min.)	30	30
	Adsorbent dosage (gm)	0.6	0.3
	pH	4	4
Cu^2+^	Initial concentration (ppm)	100	300
	Contact time (min.)	30	30
	Adsorbent dosage (gm)	–	0.3
Cd^2+^	pH	–	3
	Initial concentration (ppm)	–	200
	Contact time (min.)	–	30

### 3.3.1. Effect of adsorbent dosage

Adsorbent dosage is a critical factor that influences adsorption efficiency and determines the amount of adsorbent required for effective pollutant removal^49^. As shown in Figs. 6a–c, increasing the dosage of PD or SBPD leads to a higher removal percentage of metal ions from solution.

The optimum dosage for PD was found to be 0.4 g, which removed approximately 347 ppm Pb²⁺ (99%) from an initial concentration of 350 ppm. A dosage of 0.6 g of PD adsorbed 96 ppm Cu²⁺ from an initial concentration of 100 ppm (Table 3).

For SBPD, the optimum dosage was 0.2 g for the removal of 1000 ppm Pb²⁺ from an initial concentration of 1000 ppm. Additionally, 0.3 g of SBPD removed 300 ppm Cu²⁺ from an initial concentration of 300 ppm and 198 ppm Cd²⁺ (99%) from an initial concentration of 200 ppm (Table 3).

### 3.3.2. Effect of pH

**Fig. 7:**
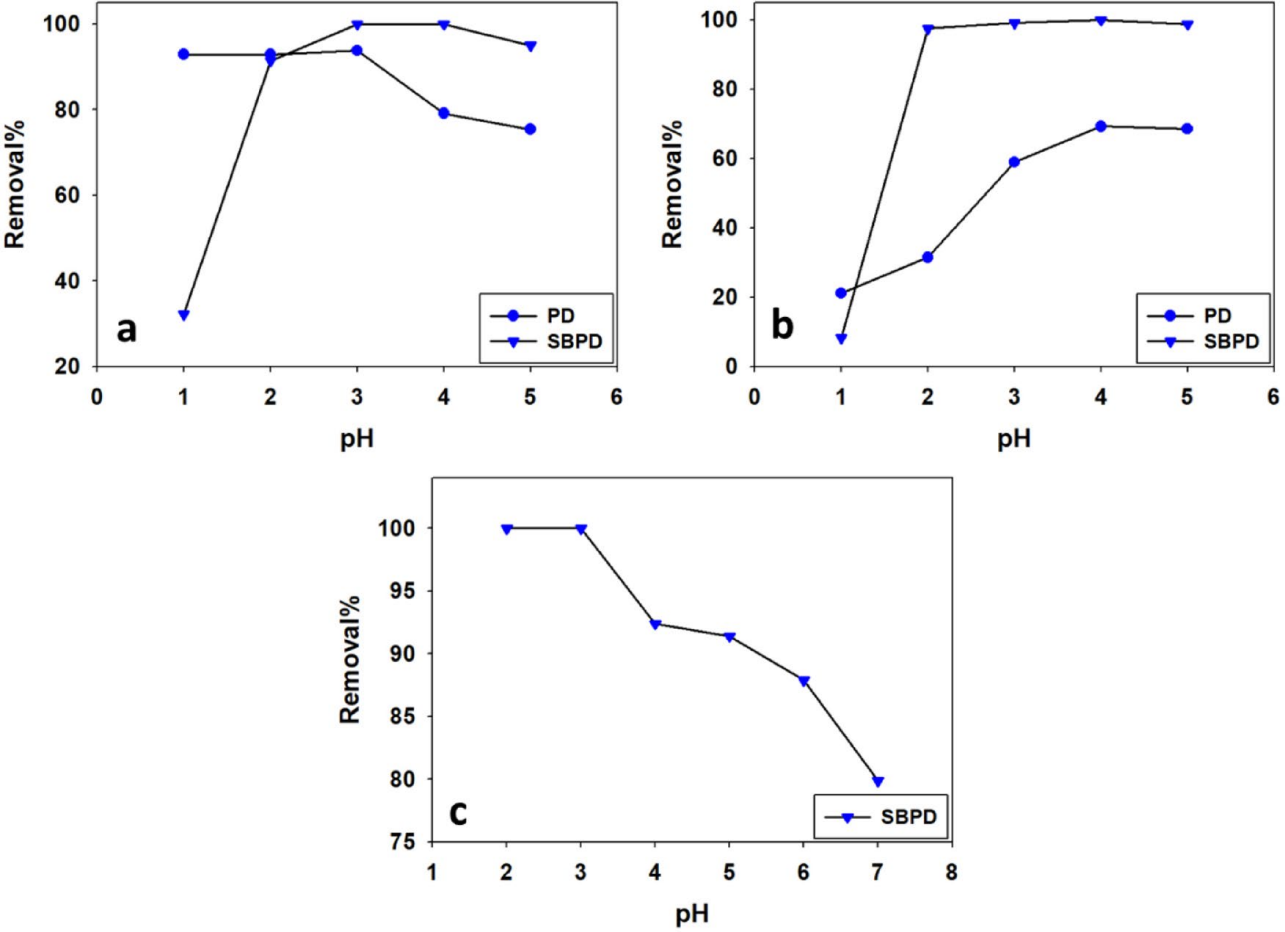
The optimum pH for removal of Pb^2+^ (**a**), Cu^2+^ (**b**) onto PD and SBPD, and Cd^2+^ onto SBPD (**c**).

pH is a critical factor influencing adsorption because it significantly affects both the ionization state of metal ions and the surface properties of the adsorbent^50^. Figure 7 illustrates the effect of pH on the adsorption of different heavy metal ions.

At pH values above 5, hydroxide species such as Pb(OH)ⁿ²⁻ⁿ (*n* = 1, 2, 4) may form^51^. Therefore, pH values above 5 were excluded to avoid precipitation phenomena. Copper ions tend to form hydroxide species such as Cu(OH), Cu(OH)₂, Cu(OH)₃, and Cu(OH)₄ at higher pH values^52^. Similarly, cadmium species may transform into Cd²⁺ and CdOH⁺ when the pH exceeds 7^53^. Consequently, adsorption experiments were conducted within a pH range of 1–5.

The results show that increasing of pH enhanced Pb²⁺ removal with SBPD but decreased it with PD (Fig. 7a). For Cu²⁺, removal efficiency increased with increasing pH for both adsorbents (Fig. 7b). In contrast, Cd²⁺ removal slightly decreased with increasing pH (Fig. 7c).

Using PD, the optimum pH was 3, achieving 93.8% from 350 ppm Pb²⁺ removal. SBPD achieved complete removal (100%) from 1000 ppm Pb²⁺ at the same pH 3. The highest Cu²⁺ removal occurred at pH 4, reaching 69.3% of 100 ppm Cu²⁺ using PD, and 92.4% from 300 ppm using SBPD adsorbent. For Cd²⁺, the optimum pH was 3, achieving 100% of 300 ppm Cd²⁺ removal using SBPD (Table 3).

### 3.3.3. Effect of contact time

**Fig. 8:**
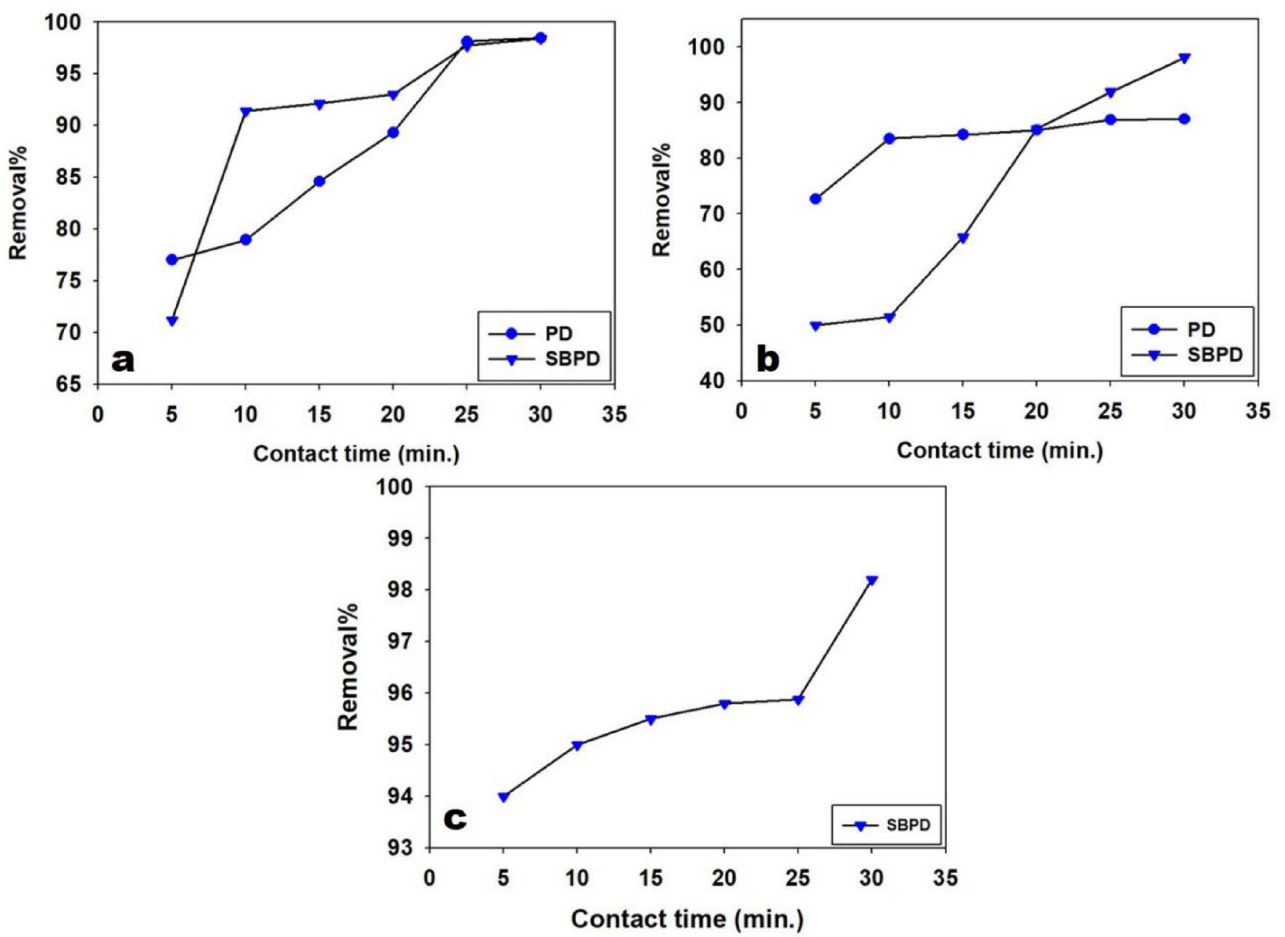
Influence of contact time on removal percentage of Pb^2+^ (**a**), Cu^2+^ (**b**), and Cd^2+^ onto SBPD (**c**).

Contact time is an important parameter in designing an economical wastewater treatment system^54^. Maximum removal of Pb²⁺ was achieved after 30 min of contact using both PD and SBPD (Fig. 8). At this time, the removal rate reached 98.4%. For Cu²⁺, the maximum removal percentages were 87% using PD and 98.1% using SBPD after 30 min. Similarly, SBPD showed optimal adsorption for Cd²⁺ after 30 min, achieving a removal rate of 98.2%.

### 3.3.4. Effect of initial concentration

**Fig. 9:**
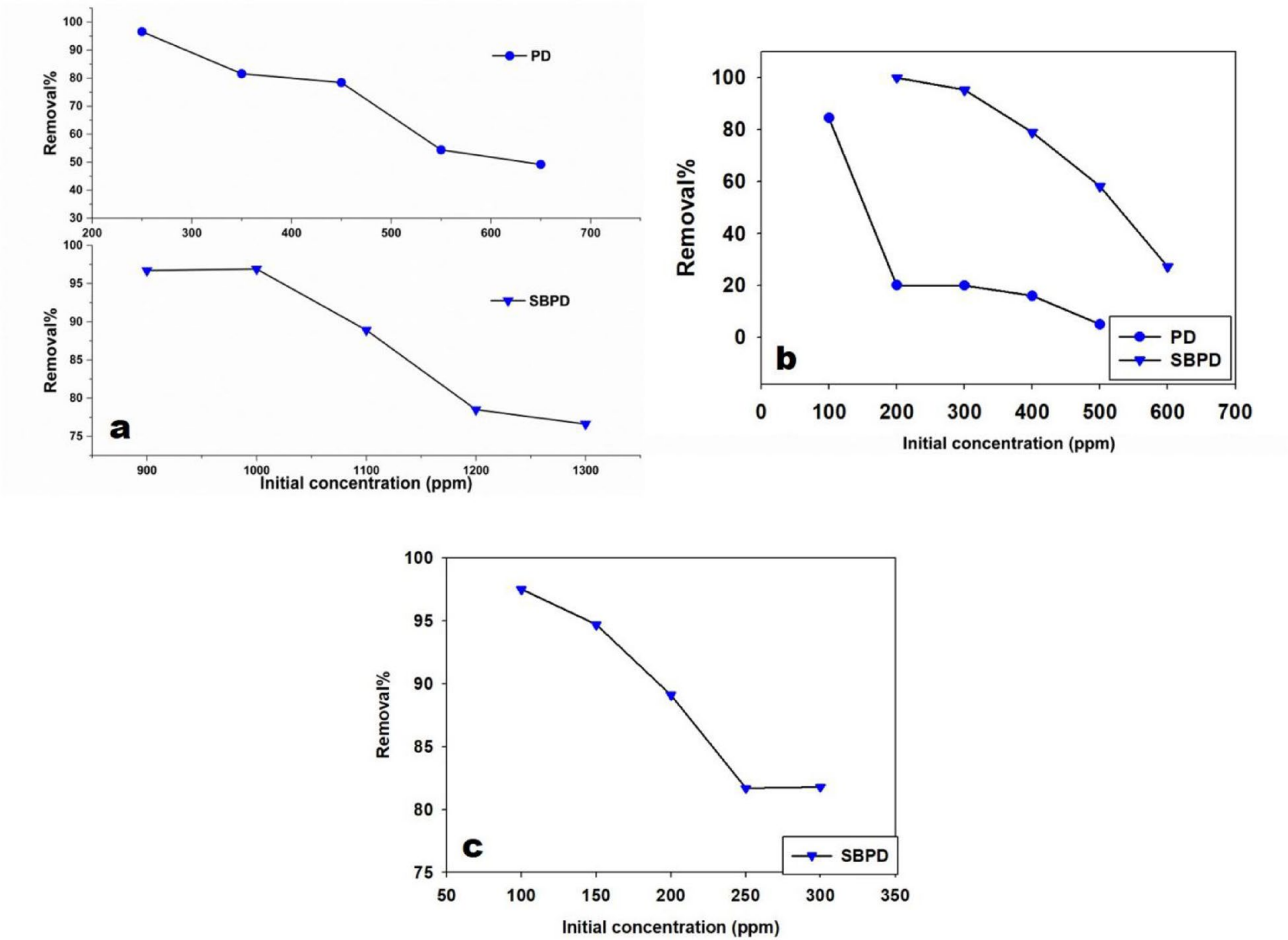
The effect of initial concentration for: (**a**) Pb^2+^, (**b**) Cu^2+^, and (**c**) Cd^2+^ onto SBPD.

The adsorption process is strongly influenced by the initial concentration of metal ions in solution^55^. As the initial concentration of Pb²⁺, Cu²⁺, and Cd²⁺ increased, the removal efficiency generally decreased due to the limited number of available adsorption sites (Fig. 9).

For PD, the maximum removal percentage of Pb²⁺ occurred at an initial concentration of 350 ppm, achieving an 82% removal rate (287 ppm removed). For Cu²⁺, the highest removal (84%) was observed at an initial concentration of 100 ppm.

For SBPD, the highest removal percentage of Pb²⁺ (97%) was achieved at an initial concentration of 1000 ppm. Cu²⁺ removal reached 95% at an initial concentration of 300 ppm, while Cd²⁺ removal reached 82% at an initial concentration of 200 ppm.

To ensure the validity of the results, parameters such as pH, adsorbent dosage, and contact time were kept constant throughout the experiments.

### 3.3.5. Co-removal of Pb^2+^, Cu^2+^, and Cd^2+^

To evaluate the effect of competitive adsorption among Pb, Cu, and Cd on the adsorption capacities of PD and SBPD, ternary system adsorption experiments were conducted under the following conditions:

1. PD adsorbent: 0.4 g dosage, initial concentration of 200 ppm for each metal ion (Pb, Cu, and Cd), contact time of 30 min, and pH 5.
2. SBPD adsorbent: 0.3 g dosage, initial concentration of 500 ppm for each metal ion (Pb, Cu, and Cd), contact time of 30 min, and pH 5.

**Fig. 10:**
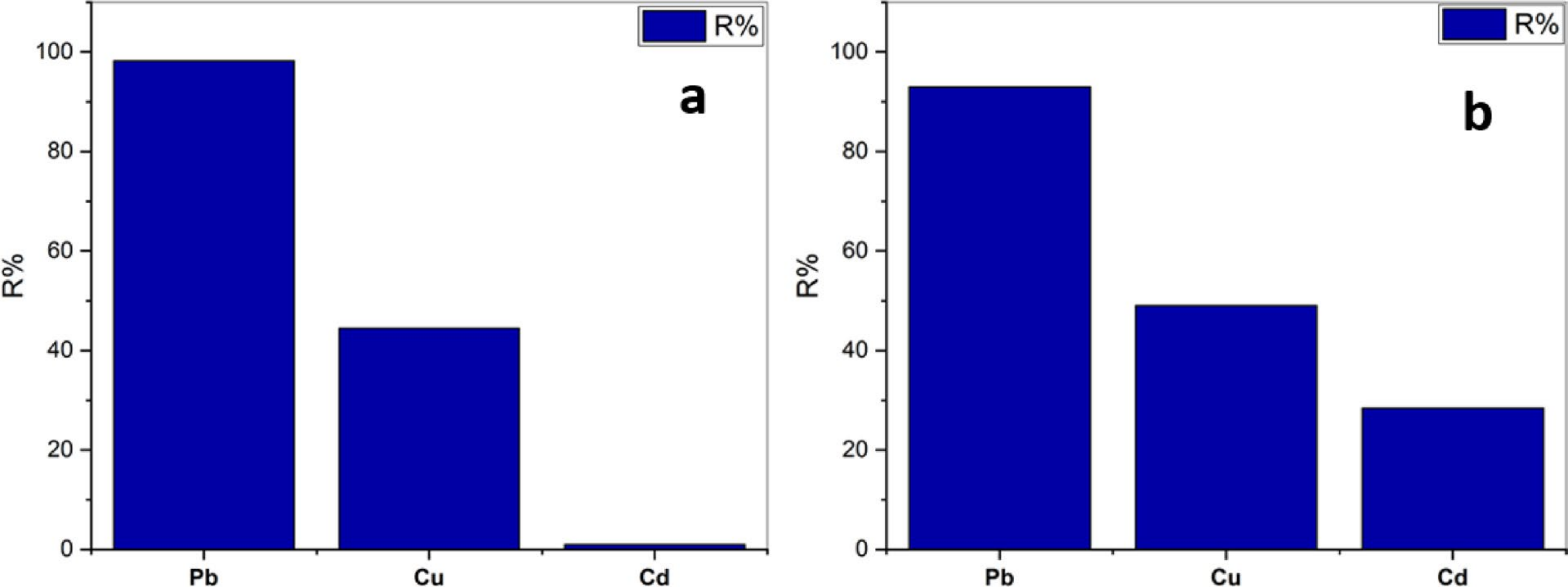
Trimetals Pb^2+^, Cu^2+^, and Cd^2+^ Adsorption onto PD (**a**) and SBPD (**b**).

Compared with the single-metal system, the removal efficiencies of Pb, Cu, and Cd decreased in the ternary system (Fig. 10). This reduction may be attributed to increased electrostatic repulsion and competition among metal ions for the available adsorption sites, which suppresses metal uptake in multi-component systems^56^.

According to the results presented in Fig. 9a, PD exhibits a higher affinity toward Pb²⁺, following the order Pb²⁺ > Cu²⁺ > Cd²⁺. Thus, Pb acts as the dominant competitor due to its stronger adsorption tendency. Similarly, the sodalite - based material shows the highest adsorption capacity for Pb²⁺, followed by Cu²⁺ and Cd²⁺, confirming the same adsorption sequence (Pb²⁺ > Cu²⁺ > Cd²⁺) as shown in Fig. 10b.

The affinity of the sorbent toward heavy metal ions depends on several factors, including polarizability, ionization potential, electronegativity, ionic radius, and softness of the metals^57^. Pb exhibits relatively high electronegativity, a lower hydrated radius, and a favorable electronic structure, which leads to preferential adsorption compared with Cd²⁺ and Cu²⁺^58^. Fan et al^59^. also reported that the electronegativity of metal ions plays a key role in selective adsorption on NaX zeolite. Metal ions with higher electronegativity can more strongly attract the electron clouds of negatively charged oxygen atoms in the zeolite framework.

Therefore, the selective adsorption capacity of PD and SBPD follows the order Pb²⁺ > Cu²⁺ > Cd²⁺, which can be attributed to differences in hydration energy and electronegativity^59^. Pb²⁺ possesses the lowest hydration energy (1502 kJ/mol) and relatively higher electronegativity (2.33) compared with Cu²⁺ and Cd²⁺, resulting in stronger adsorption.

### 3.3.6. Adsorption isotherm

Adsorption isotherms describe the equilibrium relationship between adsorbent and adsorbate during the adsorption process^60^. Several models have been developed to explain the interaction between solid adsorbents and adsorbed molecules.

The Langmuir isotherm model assumes a homogeneous adsorbent surface with identical adsorption sites and uniform adsorption energy. Under these conditions, adsorption occurs as a monolayer through a chemisorption mechanism^61^. The linear form of the Langmuir equation is expressed as represented by “Equation (1)”:

1 $$\frac{{{\mathrm{C}}_{e} }}{{{\mathrm{q}}_{e} }} = \frac{1}{{{\mathrm{b}}\;{\mathrm{q}}_{{\max }} }} + \frac{{{\mathrm{C}}_{e} }}{{{\mathrm{q}}_{{\max }} }}$$

To determine the values of q_max_ and b in the Langmuir isotherm model, a plot of (C_e_/q_e_) versus C_e_ can be used. In this context, q_e_ represents the amount adsorbed in mg/g, b indicates the equilibrium constant of adsorption, q_max_ denotes the maximum adsorption capacity in mg/g, and C_e_ is the equilibrium concentration in ppm. Additionally, “Equation (2)” defines a dimensionless constant known as the separation factor (R_L_)^62^.

2 $${\mathrm{R}}_{{\mathrm{L}}} = \frac{1}{{\left( {1 + {\mathrm{b}}\,{\mathrm{C}}_{i} } \right)}}$$

The separation factor (R_L_) is an important parameter for characterizing the adsorption process. If R_L_ is greater than 1, the adsorption is considered unfavorable. A value of R_L_ equal to 1 indicates a linear adsorption process. When R_L_ falls between 0 and 1, the adsorption is deemed favorable. Lastly, a R_L_ value of 0 signifies an irreversible adsorption process^62^.

The Freundlich isotherm model is used to describe adsorption processes that occur on heterogeneous surfaces with active sites exhibiting varying energy levels, accounting for multilayered adsorption and equilibrium^63^. The linear form of the Freundlich isotherm model is given by “Equation (3)”^64,65^:

3 $$\log \;q_{e} = \log \;K_{f} + \frac{1}{n}\log \,C_{e}$$

A plot of log qe versus log Ce provides the constants Kf (adsorption capacity) and n (adsorption intensity) from the intercept and slope, respectively^64,65^.

Figures 12 and 13 show the Langmuir and Freundlich isotherm plots, while Table 4 summarizes the calculated constants. A correlation coefficient (R²) close to 1 indicates a strong agreement between the experimental data and the model.

**Fig. 11:**
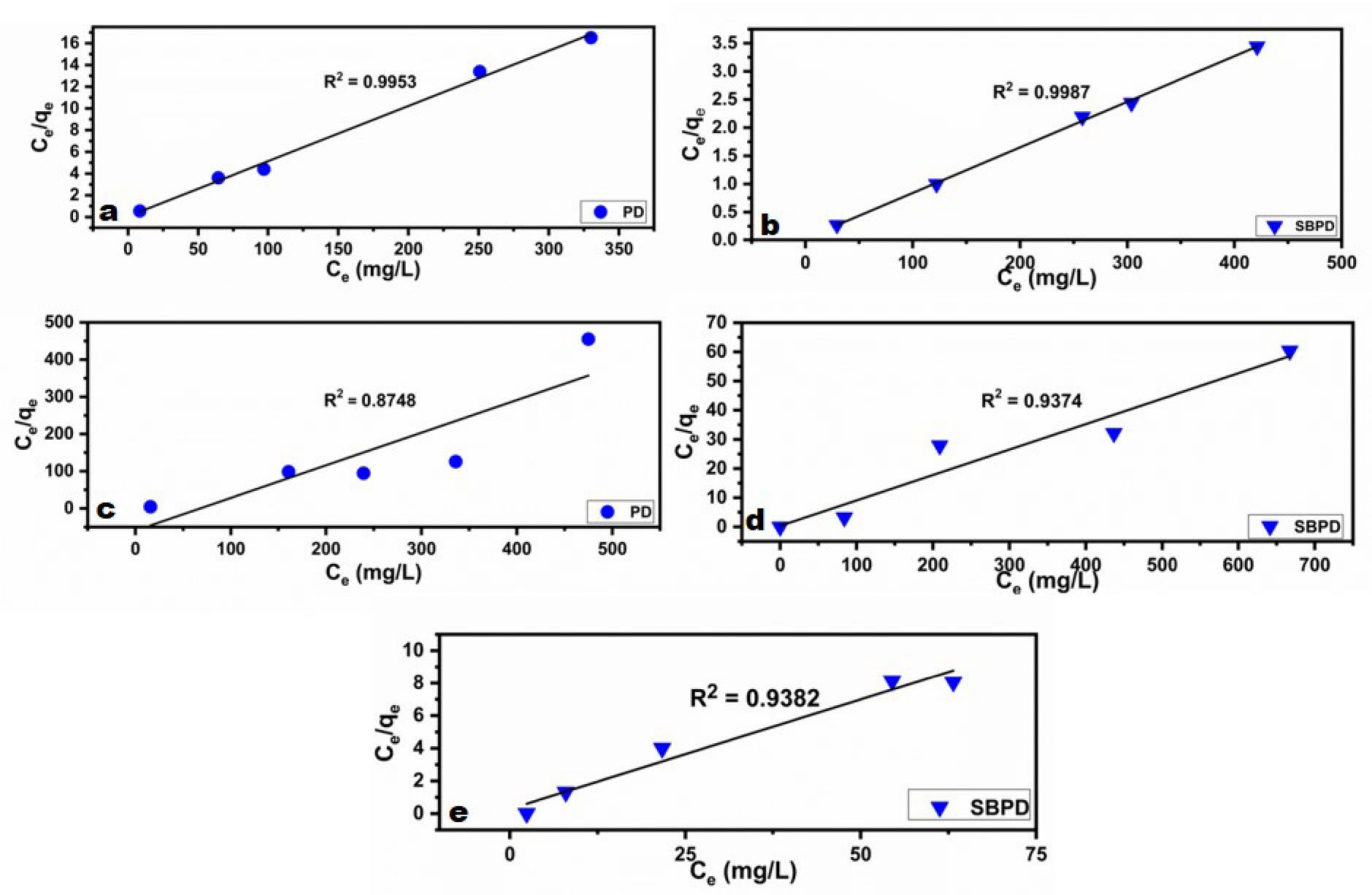
Langmuir isotherm plots for the adsorption of Pb^2+^ and Cu^2+^ onto PD (**a** and **c**), and Pb^2+^, Cu^2+^ and Cd^2+^ onto SBPD (**b**, **d**, **e**) respectively.

**Fig. 12:**
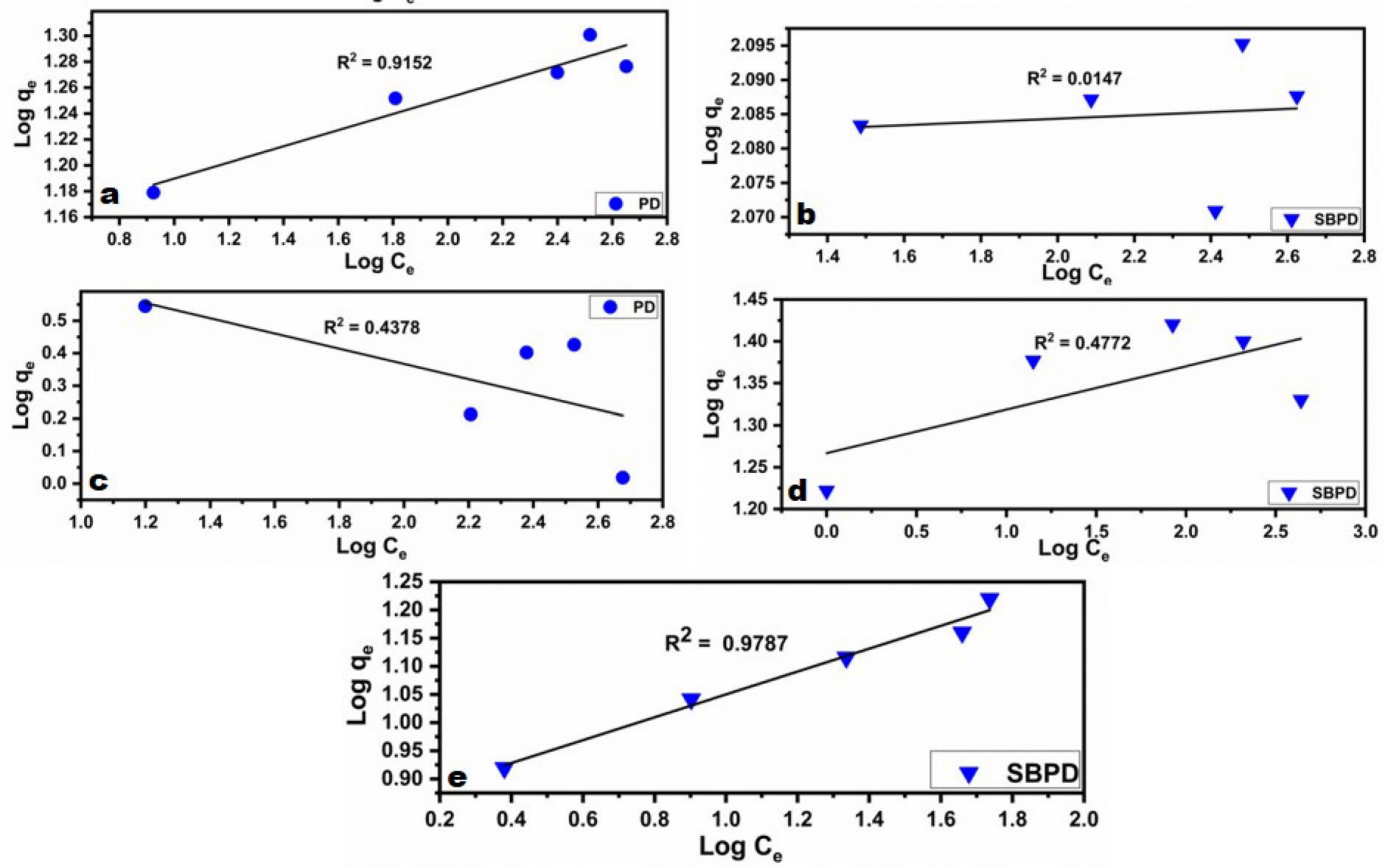
Freundlich isotherm plots for the adsorption of Pb^2+^ and Cu^2+^ onto PD (**a** and **c**), and Pb^2+^, Cu^2+^ and Cd^2+^ onto SBPD (**b**, **d**, **e**) respectively.

**Table 4:** Langmuir and Freundlich isotherml models parameters and correlation coefficients for Pb^2+^, Cu^2+^, and Cd^2+^ adsorption onto PD and SBPD.

Heavy metal	Model	Parameters	Phosphatic dolomite (PD)	Sodalite (SBPD)
Pb^2+^	Langmuir	q_max_ (mg/g)	19.7	123.5
		K_L_ (b)	0.8	0.27
		R_L_	0.005 − 0.002	0.004–0.002
		R^2^	0.9953	0.9987
	Freundlich	K_f_	13.41	120.1
		n	16.02	416.7
		R^2^	0.9152	0.0147
Cu^2+^	Langmuir	q_max_ (mg/g)	2.74	11.5
		K_L_ (b)	0.03	0.27
		R_L_	0.25 − 0.05	0.018–0.004
		R^2^	0.8748	0.9374
	Freundlich	K_f_	6.84	18.5
		n	−4.27	19.3
		R^2^	0.4378	0.4772
Cd^2+^	Langmuir	q_max_ (mg/g)	–	12.1
		K_L_ (b)	–	0.11
		R_L_	–	0.083–0.025
		R^2^	–	0.9382
		K_f_	–	3.3
	Freundlich	n	–	3.69
		R^2^	–	0.9787

For the adsorption of Pb²⁺ and Cu²⁺ onto PD and SBPD, the Langmuir model provided the best fit (Fig. 11a–d), suggesting mono-layer adsorption on homogeneous surfaces. Additionally, the RL values (0–1) indicate favorable adsorption.

In contrast, Cd²⁺ adsorption onto SBPD (Figs. 11e and 12e; Table 4) showed R² = 0.94 for the Langmuir model, with a maximum adsorption capacity (Q_max_) of 12.1 mg/g and RL values between 0.0083 and 0.0023, confirming favorable adsorption.

For the Freundlich model, the correlation coefficient was R² = 0.98, with a heterogeneity factor *n* = 3.39 (1/*n* = 0.27), indicating favorable adsorption.

These results suggest that both Langmuir (R² = 0.94) and Freundlich (R² = 0.98) models describe Cd²⁺ adsorption onto SBPD, although the Freundlich model provides a slightly better fit. Hamoudi et al^66^. suggested that cadmium adsorption on zeolite may occur in two stages: an initial monolayer adsorption (Langmuir) followed by multilayer adsorption (Freundlich).

The difference in isotherm fitting for Pb²⁺ and Cu²⁺ (Langmuir) compared with Cd²⁺ (Freundlich) may be explained by the lower KL value of Cd²⁺ (0.11) relative to Pb²⁺ and Cu²⁺ (0.27), indicating a weaker Cd²⁺–SBPD interaction. This weaker bonding may promote lateral interactions among adsorbed Cd²⁺ ions, leading to adsorption on heterogeneous surfaces.

### 3.3.7. Kinetic studies

Adsorption kinetics are important for determining the minimum contact time required for efficient adsorption. In this study, the adsorption mechanisms of Pb²⁺, Cu²⁺, and Cd²⁺ onto PD and SBPD were investigated using the pseudo-first-order, pseudo-second-order, and intra-particle diffusion models^67–69^.

The pseudo-first-order kinetics is described by the following “equation (4)”:

4 $$\log \;\left( {q_{e} - q_{t} } \right) = \log \,q_{e} - \frac{{\left( {K_{1} t} \right)}}{{2.303}}$$

In this model, the rate constant k_1_ (mg/g min) represents the rate of adsorption. Here, q_e_ (mg/g) denotes the quantity of heavy metal ions adsorbed at equilibrium, while q_t_ (mg/g) indicates the amount adsorbed at a specific time t.

To apply the pseudo-second-order “equation (5)” is utilized in the given form:

5 $$\:\frac{{t}}{{c{q}}_{{t}}}=\:\frac{1}{\left({{k}}_{2\:}{{q}}_{{e}}^{2}\right)}+\frac{{t}}{{{q}}_{{e}}}$$

The second-order rate constant, k_2_ (mg/g min), can be obtained from the plot of t/q_t_ versus t. Additionally, “equation (6)” can be used to calculate the initial adsorption rate h (mg/g min)^67^.

6 $$h = k_{2} .q_{e} ^{2}$$

**Fig. 13:**
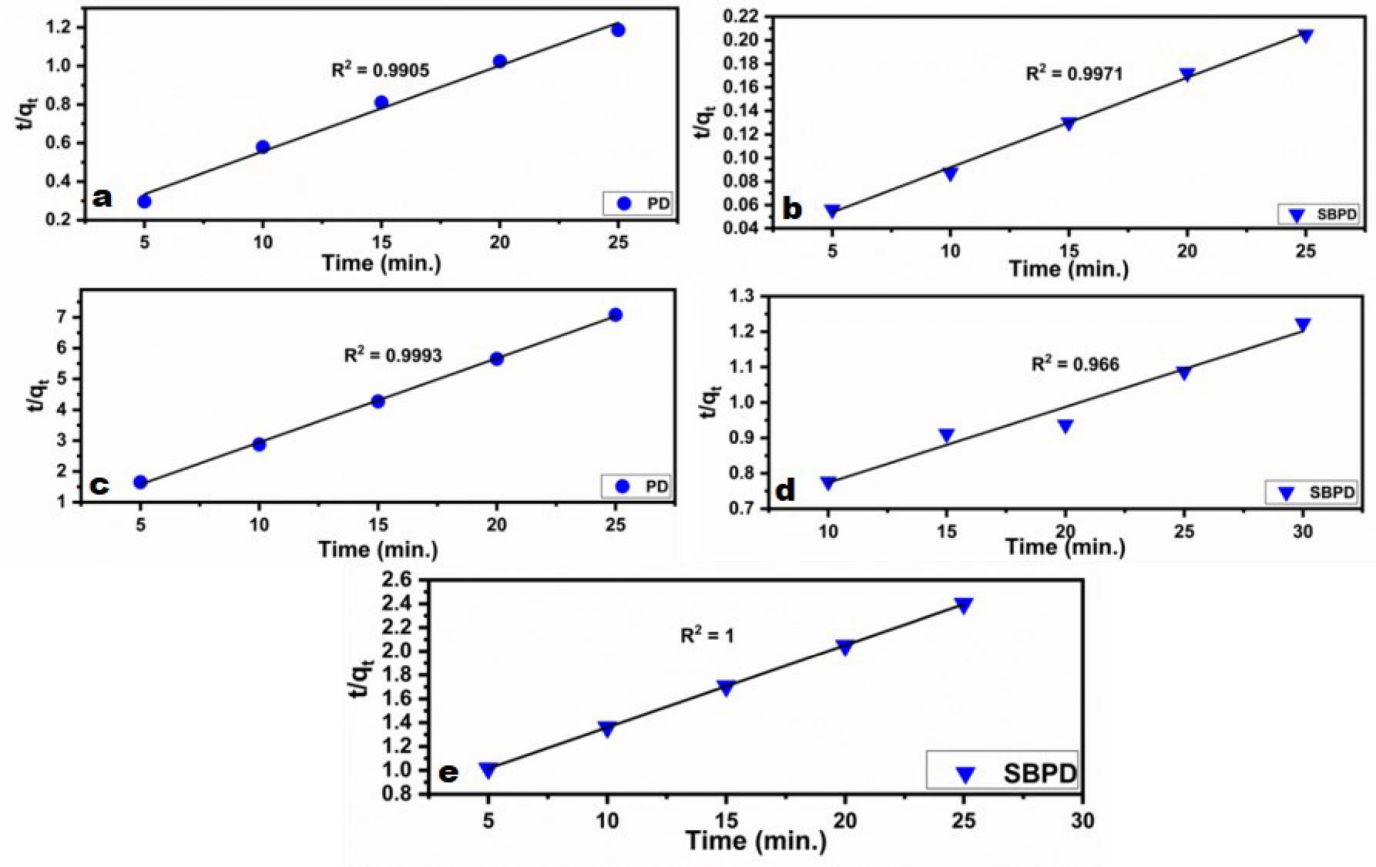
Pseudo-second-order kinetic plots for the heavy metal ions Pb^2+^and Cu^2+^ (**a**, **c**) onto PD, and Pb, Cu^2^and Cd^2+^(**b**, **d**, **e**) onto SBPD.

Based on the data presented in, the pseudo-first order model is not fit to interpret the kinetics of Pb, Cu and Cd ions adsorption onto PD and SBPD. In the other hand, it is evident from Figure (13) that the pseudo-second-order kinetic model offers a superior fit. Table (5) provides the relevant parameters and regression coefficient (R^2^) for the analyzed data. The obtained regression coefficient (R^2^) for the plot exceeds 0.96, indicating a high degree of accuracy in the model fit. As a result, it can be concluded that the adsorption of heavy metals (Pb^2+^, Cu^2+^, Cd^2+^) onto PD and SBPD follows a chemisorption process^70^.

The intra-particle diffusion (IP) model is frequently used to analyze the rate-limiting step in adsorption processes. In many adsorption scenarios, the uptake of adsorbates is proportional to the square root of time (t^1/2^)^71^. The intra-particle diffusion model can be represented by the following “equation (7)”^71^:

7 $$q_{t} = k_{{IPD}} t^{{1/2}} + C$$

where K_IPD_ is the intra-particle diffusion constant (mg/min^1/2^. g), and C represents the thickness of the boundary layer (intercept). The high value of C confirmed that surface adsorption is highly effective in the rate-limiting step.

According to Wang and Guo^72^ the intraparticle difusion model is interpreted by three mass transfer processes: the first is the external diffusion (or film diffusion) includes the transfer of adsorbate in the liquid film around the adsorbent, the second is the internal diffusion (or intraparticle diffusion) includes the transfer of adsorbate in the pores of the adsorbent and the third is the adsorption onto the active sites.

When applying the intraparticle diffusion model the following features must be considered : if the model line passes through (0, 0), the adsorption is dominated by the intraparticle diffusion, if not, it is controlled by the multiple processes [72. Also, Multi-linearity feature in the intraparticle diffusion model plot indicates that adsorption occurs in multiple, successive stages rather than a single process. Typically, this reveals three distinct steps: boundary layer diffusion (external surface adsorption), pore diffusion (gradual adsorption), and final equilibrium^73^.

**Fig. 14:**
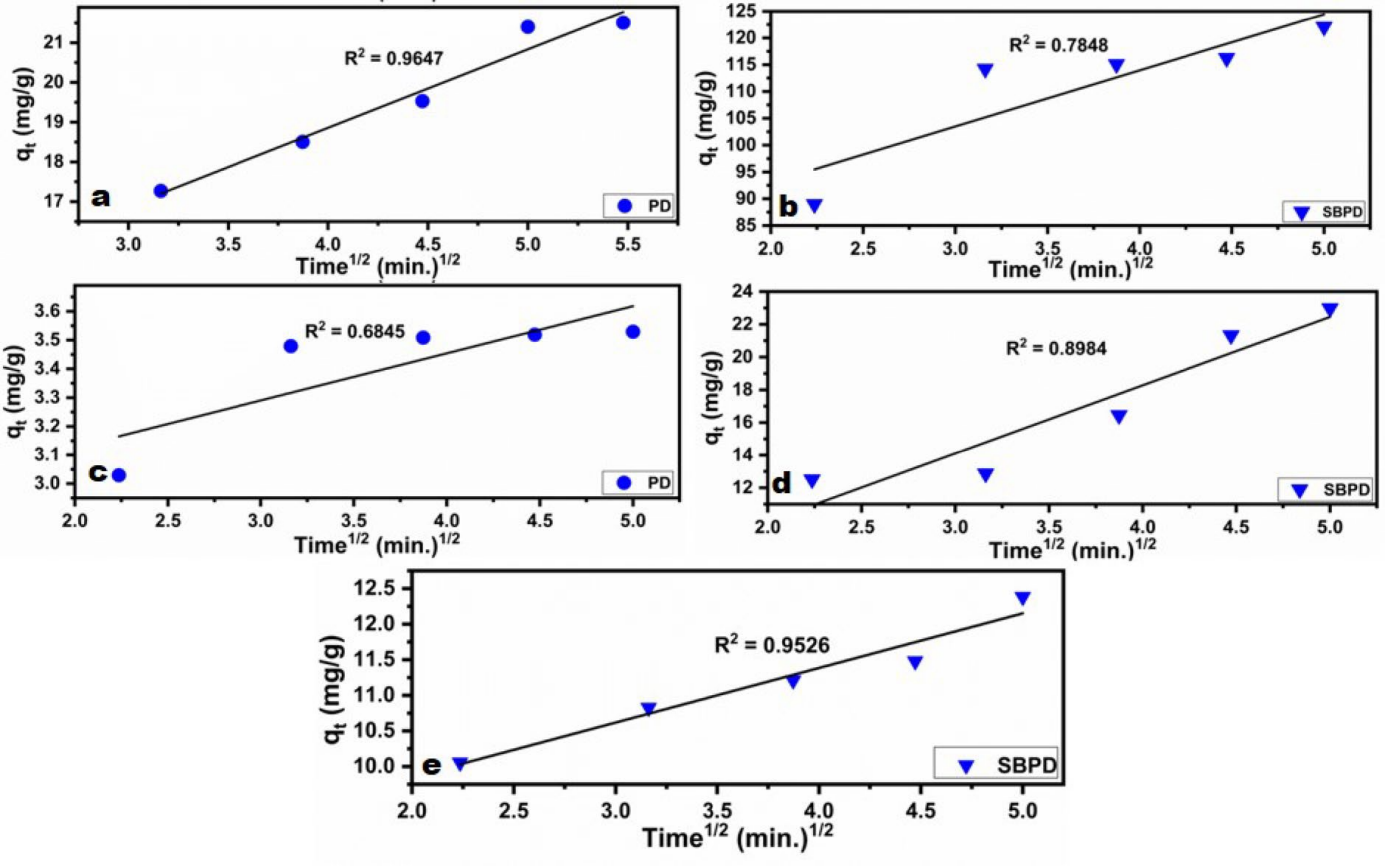
Intra-particle diffusion kinetic model plots for the heavy metal ions Pb^2+^and Cu^2+^ (**a**, **c**) onto PD, and Pb, Cu^2^and Cd^2+^(**b**, **d**, **e**) onto SBPD.

The Intra-particle diffusion model was applied in the present study to inspect the diffusion mechanism of heavy metals adsorption onto PD and SBPD. The results are given in Fig. (14) and Table (6). The plots of q_t_ vs. t^1/2^ show the model line is not passes through (0,0) point which indicates that the adsorption in the present study is controlled by the multiple processes.

It is proved from the results give in Figs. 13and 14 as well as Tables 4 and 5 that pseudo-second order as well as intra-particle are fitting with the adsorption kinetics of Pb, Cu and Cd ions onto PD and SBPD.

**Table 5:** Parameters of the studied pseudo-first-order and pseudo-second-order kinetic models of Pb, Cu, and Cd adsorption onto PD and SBPD.

**Heavy metal**	**Model**	**Parameters**	**Phosphatic dolomite (PD)**	**Sodalite (SBPD)**
Pb^2+^	Pseudo-First-order	K_1_ (mg/g min)	–	0.16
		q_e_ (mg/g)	–	61.1
		R^2^	–	0.8578
	Pseudo-Second-order	K_2_ (mg/g min)	0.017	0.004
		q_e_ (mg/g)	22.5	131.6
		h (mg/g min)	8.93	63.7
		R^2^	0.9905	0.9971
Cu^2+^	Pseudo-First-order	K_1_ (mg/g min)	–	0.13
		q_e_ (mg/g)	–	33.6
		R^2^	–	0.883
	Pseudo-Second-order	K_2_ (mg/g min)	0.35	0.0008
		q_e_ (mg/g)	3.66	46.7
		h (mg/g min)	4.68	1.8
		R^2^	0.9993	0.966
Cd^2+^	Pseudo-First-order	K_1_ (mg/g min)	–	0.026
		q_e_ (mg/g)	–	0.44
		R^2^	–	0.96
	Pseudo-Second-order	K_2_ (mg/g min)	–	1.15
		q_e_ (mg/g)	–	8.01
		h (mg/g min)	–	73.5
		R^2^	–	1

**Table 6:** Parameters of intraparticle diffusion model of Pb, Cu and Cd adsorption onto PD and SBPD.

Heavy metal	Model	Parameters	Phosphatic dolomite (PD)	Sodalite (SBPD)
Pb^2+^	Intra- Particle diffusion	K_IPD_ (mg/min^1/2^.g)	1.98	10.5
		C	10.95	72.06
		R^2^	0.9647	0.7848
Cu^2+^	Intra- Particle diffusion	K_IPD_ (mg/min^1/2^.g)	0.16	4.17
		C	2.8	1.6
		R^2^	0.6845	0.8984
Cd^2+^	Intra- Particle diffusion	K_IPD_ (mg/min^1/2^.g)	–	0.08
		C	–	7.6
		R^2^	–	0.9526

## 3.4. Conclusion

Calcined phosphatic dolomite (CPD) and black shale waste materials from the Abu Tartur phosphate mine were successfully utilized to synthesize a sodalite-based material (SBPD). These materials were evaluated for their ability to remove heavy metals (Pb²⁺, Cu²⁺, and Cd²⁺) from aqueous solutions.

The optimum conditions for PD adsorption were a dosage of 0.4 g for Pb²⁺ removal (347 ppm from 350 ppm) and 0.6 g for Cu²⁺ removal (96 ppm from 100 ppm). The optimum pH ranged from 3 to 4 with a contact time of 30 min.

For SBPD, a dosage of 0.2 g removed 1000 ppm Pb²⁺ completely, while 0.3 g removed 300 ppm Cu²⁺ and 198 ppm Cd²⁺ from aqueous solutions containing 300 ppm Cu²⁺ and 200 ppm Cd²⁺, respectively. The optimal conditions were pH values between 3 and 4 with a contact time of 30 min.

The adsorption affinity of metal ions followed the order: Pb²⁺ > Cu²⁺ > Cd²⁺ for both PD and SBPD, which can be attributed to differences in hydration energy and electronegativity.

Adsorption isotherm analysis revealed that the Langmuir model best described the adsorption of Pb²⁺ and Cu²⁺ onto both PD and SBPD, indicating monolayer adsorption on homogeneous surfaces. In contrast, the Freundlich model provided a better fit for Cd²⁺ adsorption on SBPD, suggesting multilayer adsorption on heterogeneous surfaces.

Kinetic studies demonstrated that the pseudo-second-order model and intra-particle diffusion models are best described the adsorption behavior.

Overall, SBPD exhibited significantly higher adsorption efficiency and greater potential than raw phosphatic dolomite (PD) for the removal of toxic metal ions from contaminated water.

## Data Availability

The data and chemical analysis that present in the research article are available with the corresponding author (Moneim) in reasonable request.
